# Crystalline κ-light-chain proximal tubulopathy in Waldenström macroglobulinemia with an IgA nephropathy pattern: a case report and systematic review

**DOI:** 10.3389/fimmu.2026.1942324

**Published:** 2026-10-15

**Authors:** Jun Zhang, Xing Chen, Lijing Yao, Tiekun Li, Xuejuan Sun, Xiuhua Xu, Peijin Bai, Leping Shao, Juan Pei

**Affiliations:** 1 Department of Nephrology, The First Affiliated Hospital of Xiamen University, School of Medicine, Xiamen University, Xiamen, China; 2 The School of Clinical Medicine, Fujian Medical University, Fuzhou, China; 3 Nanjing Kingmed Center for Clinical Laboratory, Nanjing, Jiangsu, China

**Keywords:** case report, IgA nephropathy pattern, light chain proximal tubulopathy, MPO-ANCA, Waldenström macroglobulinemia

## Abstract

Light-chain proximal tubulopathy (LCPT) is a rare renal manifestation of monoclonal gammopathy that is exceptionally uncommon in Waldenström macroglobulinemia (WM). We report a case of crystalline κ-LCPT in WM and conduct a literature review of WM-associated LCPT and crystal-storing histiocytosis (CSH). A 65-year-old man with a 4-year history of intermittent hematuria and proteinuria developed progressive renal dysfunction. Before admission, myeloperoxidase (MPO)-anti-neutrophil cytoplasmic antibody (ANCA) was strongly positive by qualitative immunoblotting, initially raising concern for ANCA-associated vasculitis. After admission, repeat testing on a separate sample showed only modest MPO-ANCA elevation by chemiluminescence, whereas proteinase 3 (PR3)-ANCA and both P-ANCA and C-ANCA by indirect immunofluorescence were negative; serum immunoglobulin M (IgM) was markedly elevated, and monoclonal IgM-κ was identified in serum and urine. Kidney biopsy demonstrated crystalline κ-LCPT, with κ-predominant light-chain staining in proximal tubular epithelial cells and organized intracytoplasmic crystals on electron microscopy. Concurrent glomerular findings showed an immunoglobulin A (IgA) nephropathy pattern, characterized by strong mesangial IgA/C3 deposition, polytypic glomerular κ and λ staining without light-chain restriction, and corresponding electron-dense deposits. The biopsy showed no active pauci-immune necrotizing or cellular crescentic glomerulonephritis, arguing against ANCA-mediated renal injury. Crystalline material was also identified ultrastructurally within interstitial mononuclear cells, raising but not confirming CSH because histiocytic lineage was not verified by CD68/CD163. Bone marrow examination established WM/LPL with MYD88-L265P positivity. MYD88-L265P places the underlying lymphoplasmacytic clone within the canonical MYD88–interleukin-1 receptor-associated kinase (IRAK)–Bruton tyrosine kinase (BTK)–nuclear factor κB (NF-κB) signaling axis of WM. The discordant MPO-ANCA serology was considered more likely to represent non-specific/paraprotein-associated assay reactivity. Sequential BTK inhibitor therapy (ibrutinib followed by zanubrutinib, with the temporary addition of bortezomib–dexamethasone) achieved sustained hematological response and stable renal function over 49 months of follow-up. Our literature review identified seven eligible published WM-associated LCPT and/or CSH cases, yielding eight cases including the present patient. This case expands the spectrum of WM-associated renal disease by highlighting crystalline κ-LCPT as a rare clone-related tubular lesion and underscores the importance of integrated renal pathological and hematological evaluation in distinguishing clone-related injury from coexisting renal lesions.

## 1 Introduction

Waldenström macroglobulinemia (WM) is a lymphoplasmacytic lymphoma (LPL) characterized by bone marrow infiltration and monoclonal immunoglobulin M (IgM) production, accounting for approximately 1%–2% of hematological malignancies (1, 2). Renal involvement is uncommon, with a 15-year cumulative incidence of approximately 5.1% in the largest biopsy-based cohort restricted exclusively to patients with confirmed WM (3). Across four major published cohorts comprising 150 kidney biopsies of patients with WM and related IgM-secreting B-cell lymphoproliferative disorders, the pathological spectrum encompasses AL amyloidosis (14%–33%), cryoglobulinemic glomerulonephritis (7%–21%), intracapillary monoclonal deposits (3%–36%), and tubulointerstitial lesions (14%–51%) (3–6). Light-chain proximal tubulopathy (LCPT)—characterized by intracellular accumulation of monoclonal light chains within proximal tubular epithelial cells—was identified in only 4 of these 150 biopsies (2.7%), and crystal-storing histiocytosis (CSH) was identified in only 2 (1.3%), confirming the rarity of both entities within this disease spectrum (3–6). Of these four LCPT cases, however, only one occurred in a patient with confirmed WM (3), while the remaining three (two with concurrent CSH) were described in a mixed cohort of IgM-secreting lymphoproliferative disorders in which WM status could not be confirmed at the individual-patient level (4).

LCPT comprises crystalline and non-crystalline forms, reflecting different patterns of intracellular processing and organization of monoclonal light chains within proximal tubular epithelial cells. Non-crystalline LCPT lacks organized crystals and may instead show lysosomal, granular, vacuolar, or other non-organized ultrastructural abnormalities (7). In crystalline LCPT, specific structural properties of the light-chain variable domain may confer resistance to lysosomal cathepsin-mediated degradation and promote formation of organized intracellular crystals (8–11). The crystalline form is almost exclusively κ-restricted and may require electron microscopy (EM) and antigen retrieval on pronase-digested paraffin sections for accurate diagnosis, as routine immunofluorescence is frequently false-negative (9). CSH, defined by the accumulation of crystalline immunoglobulin within tissue histiocytes, may coexist with LCPT when the crystallogenic light chain is taken up across multiple cell compartments (12, 13). Both entities are predominantly reported in multiple myeloma, with WM/LPL accounting for a small minority of published cases (12, 13).

We report a rare case of crystalline κ-LCPT in WM with a concurrent immunoglobulin A (IgA) nephropathy pattern and ultrastructural extra-tubular crystalline deposition. Myeloperoxidase (MPO)-anti-neutrophil cytoplasmic antibody (ANCA) seropositivity complicated the initial differential diagnosis, but renal pathology did not support ANCA-associated glomerulonephritis. A systematic review contextualizes this case within the published literature on WM-associated LCPT and CSH.

## 2 Case report

### 2.1 Clinical presentation

A 65-year-old man was referred to our nephrology department in March 2022 with a 4-year history of intermittent hematuria and proteinuria and progressive renal dysfunction. Renal abnormalities were first detected in 2018, when urinalysis showed 113 red blood cells/μL, 3+ blood, and 2+ protein with preserved renal function. He was treated empirically with a renin–angiotensin system inhibitor at a local clinic without kidney biopsy or nephrology referral, and subsequent urinalyses temporarily normalized. In March 2020, serum creatinine was 93 μmol/L, urine protein was negative, and urine red blood cells were 2/μL. Serum creatinine subsequently increased to 116 μmol/L in November 2020 and 157 μmol/L by August 2021, when recurrent hematuria and proteinuria were accompanied by fatigue, a 3.5-kg unintentional weight loss over the preceding 6 months, and lower-limb pain on walking. On 23 February 2022, at his first visit to our nephrology outpatient clinic, 24-h urinary protein was 1,406 mg/day, hemoglobin was 128 g/L, serum creatinine was 149 μmol/L, estimated glomerular filtration rate (eGFR) was 41.8 mL/min/1.73 m^2^, and serum albumin was 41.2 g/L. HbA1c was 5.3%, and thyroid function tests were within normal limits. Renal ultrasonography showed increased bilateral renal parenchymal echogenicity and bilateral renal cystic areas. On 3 March 2022, repeat testing showed persistent 2+ hematuria and 2+ proteinuria, 24-h urinary protein of 1,241 mg/day, and further elevation of serum creatinine to 177 μmol/L. MPO-ANCA was strongly positive by a qualitative EUROLINE immunoblot assay (EUROIMMUN, Lübeck, Germany), whereas proteinase 3 (PR3)-ANCA and anti-GBM antibodies were negative. According to the manufacturer’s performance evaluation, the anti-MPO assay had a diagnostic sensitivity of 84% and a specificity of 90% using enzyme-linked immunosorbent assay (ELISA) as the reference method. In the setting of progressive renal dysfunction with persistent hematuria and proteinuria, ANCA-associated vasculitis was considered, and the patient was admitted to our nephrology department on 18 March 2022 for further evaluation. He had no history of hypertension, diabetes mellitus, or coronary artery disease. His past medical history was notable for gastric ulcer and pulmonary emphysema with incidental pulmonary nodules; family history of kidney or hematological disease was unremarkable. He was a current smoker with an approximately 40-pack-year smoking history (approximately 20 cigarettes/day for 40 years) and reported regular alcohol consumption for more than 30 years (approximately 100 mL/day).

On 21 March 2022, repeat testing on a separate blood sample showed an MPO-ANCA level of 37.86 RU/mL (reference ≤20.0 RU/mL) and a PR3-ANCA level of 6.53 RU/mL (reference ≤20.0 RU/mL) by chemiluminescence immunoassay using HOB Biotech reagents and instrumentation (Suzhou, Jiangsu, China), whereas both P-ANCA and C-ANCA were negative by indirect immunofluorescence. PLA2R and THSD7A antibodies were negative. Serum IgM was 12.7 g/L (reference 0.4–2.3), and serum and urine immunofixation identified a monoclonal IgM-κ protein. Serum free κ light chain was 657.12 mg/L, free λ light chain was 20.38 mg/L, and the κ/λ ratio was 32.24. Complement C3 was normal; ANA and anti-dsDNA antibodies were negative. These findings prompted parallel evaluation for monoclonal gammopathy while kidney biopsy was pursued to distinguish ANCA-associated glomerulonephritis from other renal lesions. The patient's clinical course is summarized in **Table 1**.

**Table 1:** Timeline of the patient’s clinical course.

Date	Clinical/laboratory findings	Evaluation/intervention	Interpretation
2018	Urine RBCs 113/μL, blood 3+, protein 2+; serum creatinine normal	Empirical RAS inhibitor treatment at a local clinic; no kidney biopsy or nephrology referral	First documented renal abnormality
Mar 2020	Serum creatinine 93 μmol/L; urine protein negative; urine RBCs 2/μL	Routine follow-up	Apparent remission of urinary abnormalities
Nov 2020	Serum creatinine 116 μmol/L	No specific further evaluation documented	Beginning of renal function decline
Aug 2021	Hematuria/proteinuria recurred; serum creatinine 157 μmol/L; fatigue, 3.5-kg unintentional weight loss over 6 months, lower-limb pain on walking	No further diagnostic evaluation at that time	Progressive renal dysfunction with constitutional symptoms
23 Feb 2022	24-h urinary protein 1,406 mg/day; haemoglobin 128 g/L; serum creatinine 149 μmol/L; eGFR 41.8 mL/min/1.73 m^2^; HbA1c 5.3%; thyroid function normal	First nephrology visit at our hospital; renal ultrasonography showed increased bilateral parenchymal echogenicity and bilateral renal cystic areas	Clinical diagnosis of nephritic syndrome with renal insufficiency
3 Mar 2022	Persistent urine blood 2+ and protein 2+; 24-h urinary protein 1,241 mg/day; serum creatinine 177 μmol/L	MPO-ANCA strongly positive by qualitative EUROLINE immunoblot (EUROIMMUN); PR3-ANCA/anti-GBM negative	ANCA-associated vasculitis initially considered
18 Mar 2022	Admitted to nephrology	Inpatient diagnostic evaluation	Progressive kidney dysfunction prompted admission
21 Mar 2022	MPO-ANCA 37.86 RU/mL and PR3-ANCA 6.53 RU/mL by HOB Biotech chemiluminescence; P-ANCA/C-ANCA negative by IIF; PLA2R/THSD7A negative; monoclonal IgM-κ and serum free-light-chain ratio 32.24	Repeat serology and monoclonal gammopathy evaluation	Discordant MPO-ANCA serology and evidence of an IgM-κ clone
22 Mar 2022	Kidney biopsy performed	LM, IF, IHC, and EM	Crystalline κ-LCPT and an IgA nephropathy pattern; extra-tubular crystals in interstitial mononuclear cells
28 Mar 2022	Abnormal lymphoid cells 11%; CD5^−^/CD10^−^ B-cell lymphoma phenotype; MYD88-L265P positive (9.229%)	Bone marrow cytology, flow cytometry, biopsy and molecular testing	WM/LPL established by integrated hematological findings
6 Apr 2022	Splenomegaly and diffusely increased marrow metabolism	PET-CT staging	Supported systemic hematological disease
7 Apr 2022	Pre-treatment IgM peak 13.8 g/L	Ibrutinib 420 mg/day initiated	Clone-directed therapy initiated
5 May 2023 (~Month 13)	Serum creatinine 159 μmol/L; eGFR 38.4; IgM 5.43 g/L; 24-h urinary protein 521 mg/day	Switched to zanubrutinib 160 mg twice daily	Elective second-generation BTK inhibitor transition; reimbursement available
27 Jun 2024	IgM 4.22 g/L; serum creatinine 135 μmol/L; eGFR 46.5; urine protein 1+, blood 1+, RBCs 31.2/μL	Bortezomib–dexamethasone added to ongoing zanubrutinib	Added to deepen hematological response
24 Sep 2024	—	Bortezomib–dexamethasone discontinued; zanubrutinib continued	Return to zanubrutinib monotherapy
6 Jan 2025	IgM 3.42 g/L; serum creatinine 130 μmol/L; albumin 44.2 g/L	Zanubrutinib continued	Sustained hematological and renal stability
28 Apr 2026 (Month 49)	IgM 3.9 g/L; serum creatinine 139 μmol/L; eGFR 44.2 mL/min/1.73 m^2^; serum free κ/λ ratio 5.72	Zanubrutinib monotherapy continued	Clinically stable

ANCA, anti-neutrophil cytoplasmic antibody; eGFR, estimated glomerular filtration rate; EM, electron microscopy; IF, immunofluorescence; IHC, immunohistochemistry; IIF, indirect immunofluorescence; LCPT, light-chain proximal tubulopathy; LM, light microscopy; MPO, myeloperoxidase; PR3, proteinase 3; RAS, renin–angiotensin system; RBC, red blood cell; WM/LPL, Waldenström macroglobulinemia/lymphoplasmacytic lymphoma.

### 2.2 Kidney biopsy findings

Light microscopy: Across routinely stained and additional sections, 13 glomeruli were examined, of which 4 were globally sclerotic. The remaining glomeruli generally showed patent capillary loops without endocapillary hypercellularity. Mild mesangial hypercellularity and matrix expansion were present, with focal segmental areas of moderate mesangial proliferation (**Figure 1A**). Occasional segmental capillary-loop wrinkling and adhesion to Bowman’s capsule were noted, together with capsular thickening/periglomerular fibrosis and one fibrous crescent. No fibrinoid necrosis was identified. Masson trichrome staining demonstrated fuchsinophilic deposits within mesangial regions and along segmental subendothelial capillary-loop regions. Proximal tubular epithelial cells showed vacuolar and granular degeneration, focal tubular dilatation, and segmental loss of the brush border (**Figure 2A**). Tubular atrophy involved approximately 20% of the cortical area. The interstitium showed multifocal lymphocytic and mononuclear-cell infiltration with small numbers of plasma cells and neutrophils, accompanied by mild-to-moderate cortical and moderate-to-severe medullary interstitial fibrosis. Some small arteries showed intimal thickening and splitting of the elastic lamina with luminal narrowing; inflammatory cells were also present within peritubular capillaries.

**Figure 1:**
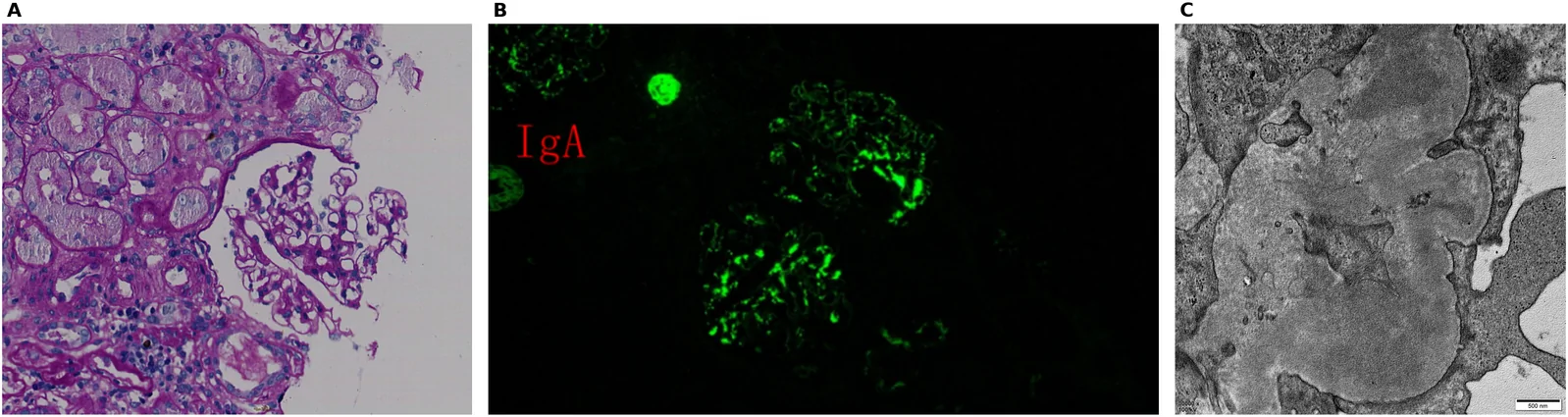
Glomerular pathological findings. **(A)** PAS staining showing mild mesangial hypercellularity and matrix expansion, with focal segmental areas of moderate mesangial proliferation. **(B)** Frozen-section IgA immunofluorescence showing strong granular and comma-shaped deposits predominantly within the mesangium and segmental capillary loops (×400). **(C)** Electron microscopy showing predominantly mesangial electron-dense deposits with occasional intramembranous deposits. The original electron microscopy scale bar is retained.

**Figure 2:**
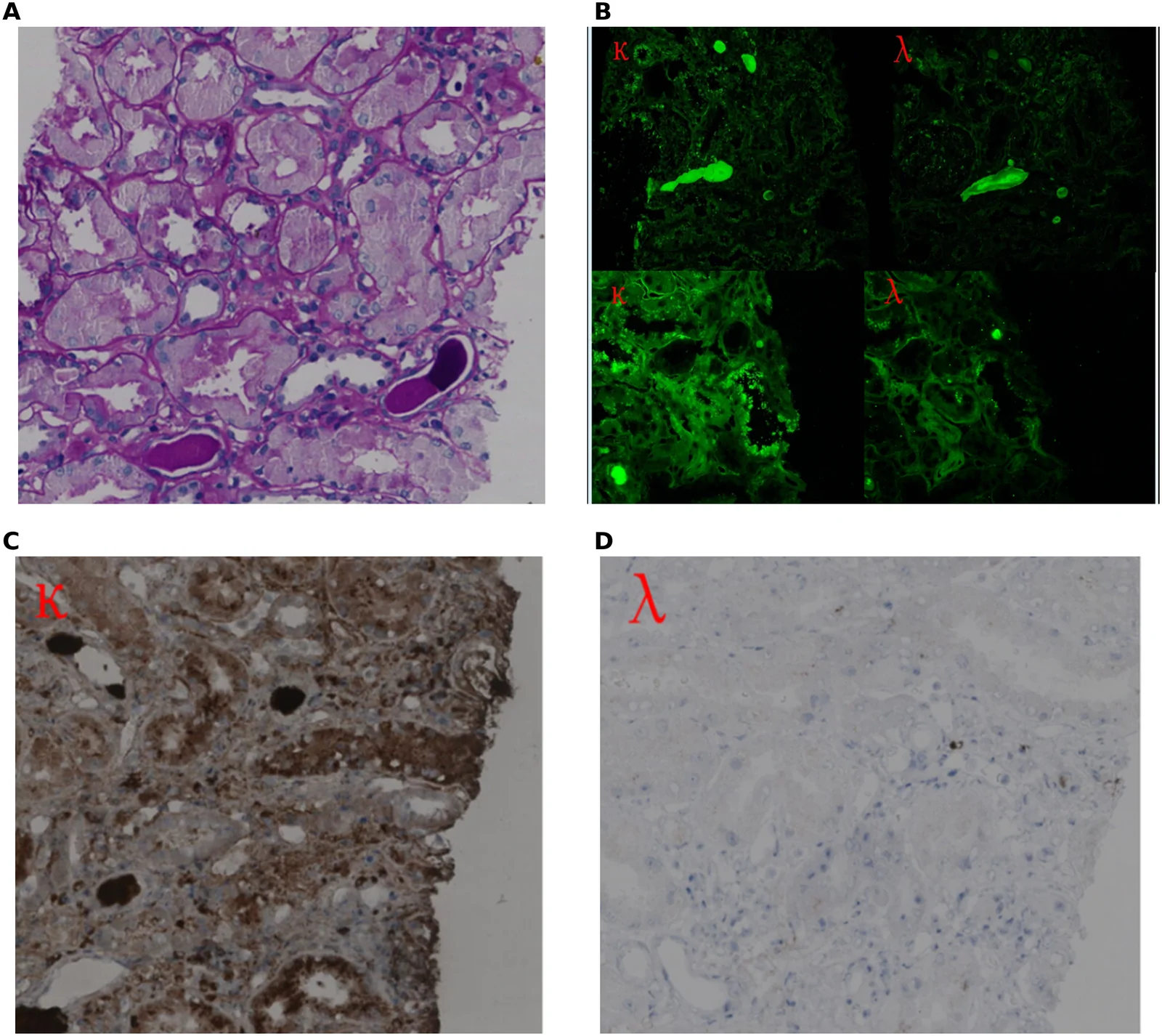
Light-microscopic and light-chain staining findings in proximal tubules and interstitium. **(A)** PAS staining showing marked swelling and pale cytoplasmic staining of proximal tubular epithelial cells, with vacuolar/granular change and segmental loss of the brush border (×200). **(B)** Pepsin-digested paraffin-section κ/λ light-chain immunofluorescence (two paired κ/λ fields) showing overall stronger κ than λ cytoplasmic staining in proximal tubular epithelial cells, with focal heterogeneity in distribution and intensity (×400). **(C, D)** κ- and λ-light-chain immunohistochemistry, respectively, showing κ-predominant cytoplasmic staining in proximal tubular epithelial cells and focal interstitial mononuclear cells, with absent or substantially weaker corresponding λ staining (DAB, ×200). Green fluorescence indicates positive immunofluorescence staining, whereas brown cytoplasmic DAB staining indicates positive immunohistochemical staining.

Immunofluorescence: Frozen-section immunofluorescence showed strong IgA (+++) and C3 (++) comma-shaped and granular deposits predominantly within the mesangium and segmental capillary loops (**Figure 1B**). IgG, IgM, and C1q were negative. On both frozen-section and pepsin-digested paraffin-section light-chain immunofluorescence, glomerular κ and λ staining was present in areas corresponding to the IgA deposits; λ was slightly stronger than κ, but the difference did not reach 2+ and was not considered evidence of light-chain restriction. Proximal tubular epithelial cytoplasm showed overall stronger κ than λ staining, with focal heterogeneity in staining intensity (**Figure 2B**).

Immunohistochemistry: A seven-marker panel on paraffin sections (CD5, CD10, CD20, CD23, MPO, κ, and λ) was performed to characterize the interstitial infiltrate and light-chain distribution. Cytoplasmic κ staining was detected in proximal tubular epithelial cells and focal interstitial mononuclear cells; corresponding λ staining was absent or substantially weaker in these compartments (**Figures 2C, D**). Scattered MPO-positive neutrophils were present within the renal interstitium.

Electron microscopy: Crystalline inclusions of variable shape and size, including organized rhomboid and rectangular profiles with sharply demarcated angular margins, were identified within the cytoplasm of proximal tubular epithelial cells, including lysosomal/membrane-bound compartments (**Figures 3A–C**). Similar rhomboid crystalline material was identified within occasional interstitial mononuclear cells (**Figure 3D**). Electron-dense immune-complex deposits were identified predominantly within the mesangium, with occasional intramembranous deposits (**Figure 1C**).

**Figure 3:**
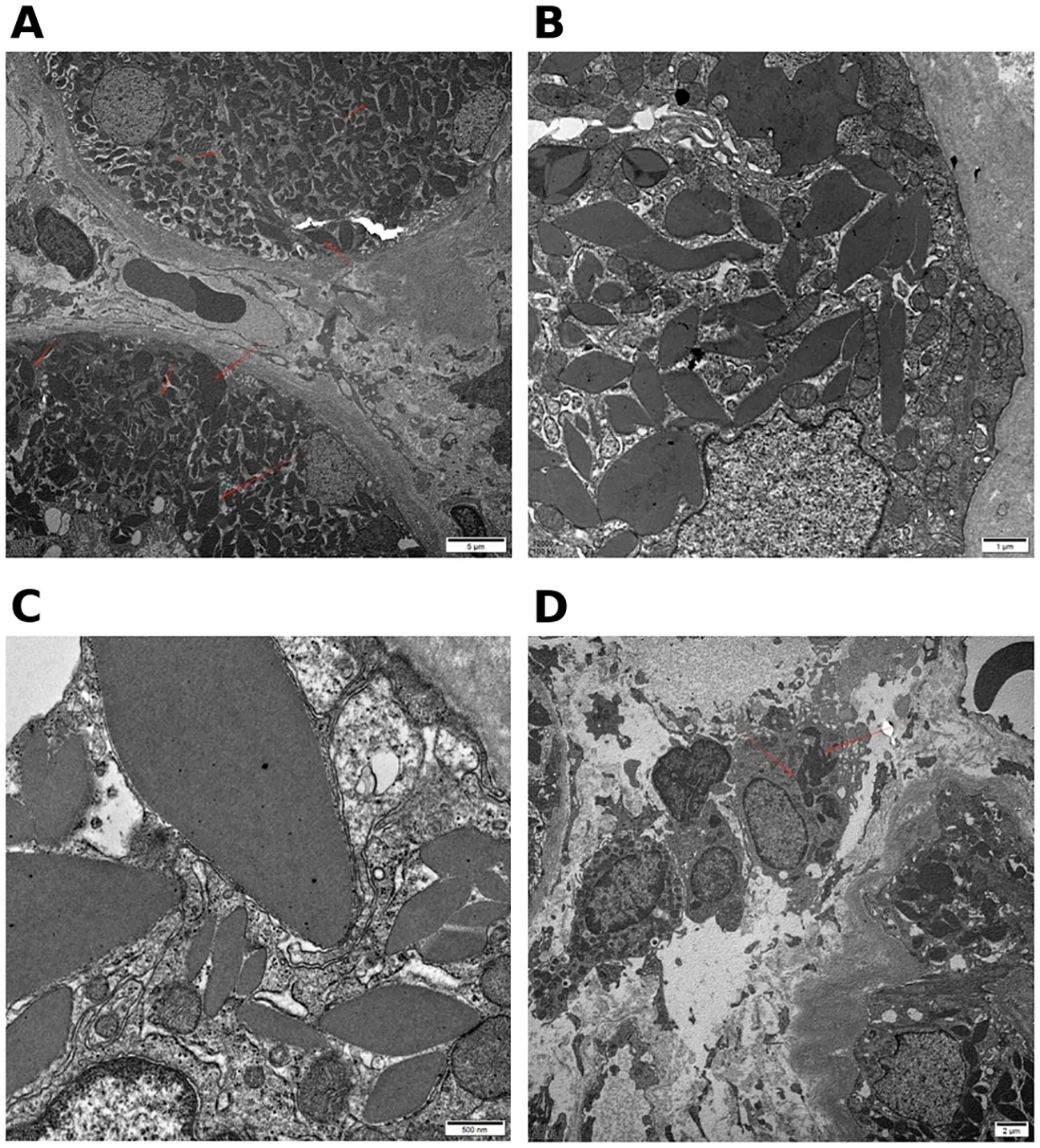
Electron microscopy findings on kidney biopsy. **(A)** Rhomboid and rectangular crystalline inclusions (red arrows) within the cytoplasm of proximal tubular epithelial cells (×3,000). **(B, C)** Higher-magnification views of the same proximal tubular crystalline inclusions shown in **(A)**, demonstrating lysosomal/cytoplasmic localization and sharply demarcated, geometrically organized profiles with straight angular margins (×12,000 and ×30,000, respectively). **(D)** A similar rhomboid crystalline inclusion (red arrow) within the cytoplasm of an interstitial mononuclear cell, anatomically distinct from the proximal tubular epithelium shown in **(A–C)** (×4,000). Original electron microscopy scale bars are retained in the lower right corner of each panel.

Integrated pathological interpretation: Taken together, the light-microscopic, immunofluorescence/immunohistochemical, and ultrastructural findings established two renal processes. First, the combination of proximal tubular injury, κ-predominant light-chain accumulation, and organized intracytoplasmic crystalline inclusions was diagnostic of crystalline κ-LCPT. Second, strong mesangial IgA and C3 deposition, polytypic glomerular κ and λ staining without light-chain restriction, and corresponding predominantly mesangial electron-dense immune-complex deposits supported an IgA nephropathy pattern. The absence of fibrinoid necrosis, cellular crescents, and a pauci-immune glomerular pattern argued against active ANCA-associated glomerulonephritis. Crystalline inclusions within occasional interstitial mononuclear cells raised the possibility of CSH but were insufficient for definitive classification.

### 2.3 Diagnosis, treatment, and outcomes

Bone marrow examination on 28 March 2022 showed 11% abnormal lymphoid cells on cytology, a CD5^−^/CD10^−^ B-cell lymphomatous phenotype on flow cytometry, CD20 positivity with CD38-positive plasma cells on immunohistochemistry, and MYD88-L265P positivity (quantitative value 9.229%), establishing LPL. Together with the circulating monoclonal IgM, these findings established the diagnosis of WM.

Ibrutinib 420 mg/day was initiated on 7 April 2022, with a rapid decline in serum IgM from a pre-treatment peak of 13.8 g/L. By 5 May 2023 (approximately month 13), serum creatinine was 159 μmol/L, eGFR was 38.4 mL/min/1.73 m^2^, IgM was 5.43 g/L, and 24-h urinary protein had decreased to 521 mg/day. Ibrutinib was then electively switched to zanubrutinib 160 mg twice daily after local reimbursement became available, given its greater selectivity and favorable tolerability profile (14); the switch was not prompted by treatment failure or a documented ibrutinib-related adverse event. To further deepen the hematological response, bortezomib–dexamethasone was temporarily added to ongoing zanubrutinib from 27 June to 24 September 2024, after which zanubrutinib monotherapy was continued.

By 6 January 2025, serum IgM had decreased to 3.42 g/L and serum creatinine had decreased to 130 μmol/L. During 2023 and 2024, our hospital performed total light-chain assays rather than serum free light-chain assays; therefore, no comparable serum free κ/λ ratio was available for those years. Serum free light-chain monitoring resumed in 2025, with κ/λ ratios of 4.42 on 17 July 2025 and 5.99 on 23 December 2025; the ratio was 5.72 at the final follow-up on 28 April 2026 (**Figure 4**). At this final follow-up (approximately month 49), serum IgM was 3.9 g/L, serum creatinine was 139 μmol/L, and eGFR was 44.2 mL/min/1.73 m^2^, indicating sustained hematological response and renal stability.

**Figure 4:**
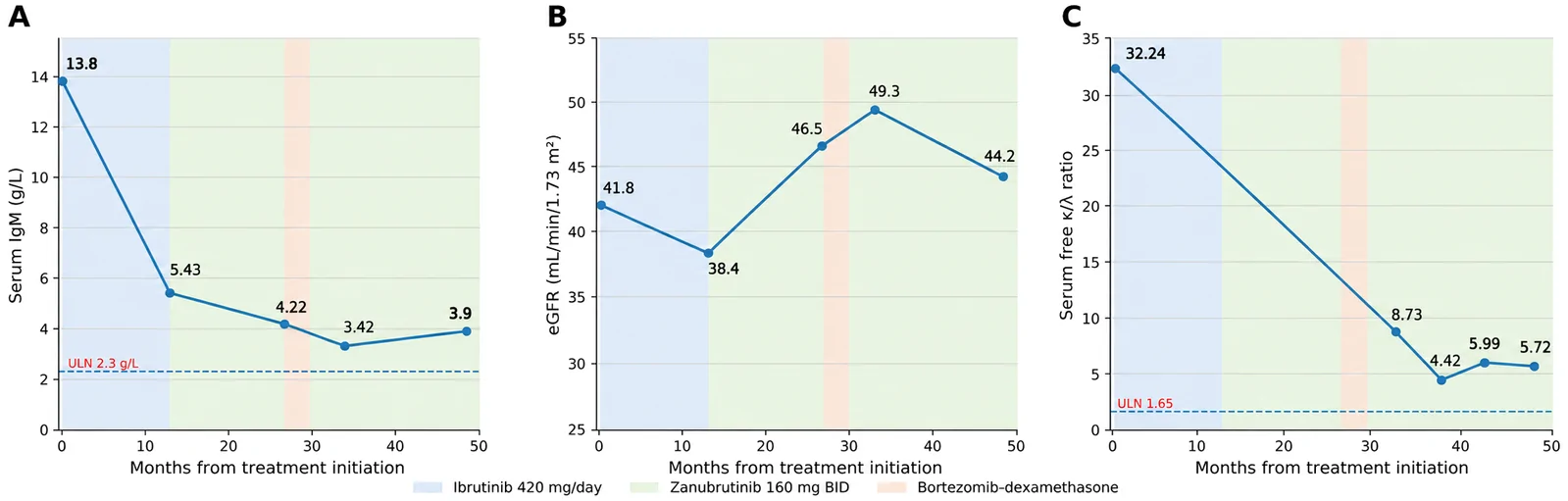
Longitudinal trends in serum IgM, eGFR, and serum free κ/λ ratio over 49 months of treatment. **(A)** Serum IgM (g/L; reference range 0.4–2.3) from pre-treatment baseline through the most recent follow-up. **(B)** Estimated glomerular filtration rate (eGFR, mL/min/1.73 m^2^) over the same period. **(C)** Serum free κ/λ ratio (reference range 0.26–1.65) at available measurement time points. Shaded bands indicate treatment phases: ibrutinib 420 mg/day (blue; initiated 7 April 2022), zanubrutinib 160 mg twice daily (green; initiated 5 May 2023), and the temporary interval during which bortezomib–dexamethasone was added to ongoing zanubrutinib (orange; 27 June–24 September 2024). The green regions therefore represent one continuous zanubrutinib course before and after the temporary add-on therapy rather than separate courses. ULN, upper limit of normal.

## 3 Literature review

### 3.1 Search strategy and case selection

#### 3.1.1 Search strategy

Three electronic databases were searched from inception to March 2026: PubMed/MEDLINE, Embase, and Web of Science, using the combined search string (“light chain proximal tubulopathy” OR “LCPT” OR “proximal tubulopathy” OR “proximal tubular disease” OR “crystalline tubulopathy” OR “crystal-storing tubulopathy” OR “crystal-storing histiocytosis” OR “crystalline histiocytosis” OR “crystalline nephropathy” OR “Fanconi syndrome”) AND (“Waldenström” OR “lymphoplasmacytic lymphoma” OR “IgM paraprotein” OR “IgM gammopathy” OR “IgM myeloma”), adapted to database-specific syntax. Reference lists of eligible full-text articles were additionally screened manually for further relevant studies. Additional relevant full-text articles identified outside the electronic database search were assessed against the same eligibility criteria, including review of supplementary patient-level data where necessary.

#### 3.1.2 Selection process

Titles/abstracts and subsequently full texts were screened independently by two reviewers against the eligibility criteria below; disagreements were resolved by discussion with a third reviewer.

#### 3.1.3 Eligibility criteria

Cases were included if they had histopathologically established renal LCPT and/or CSH in a full-text publication, a bone-marrow-confirmed diagnosis of WM or IgM-secreting LPL, and sufficient individual-level clinicopathological data for verification and extraction of key variables. Crystalline LCPT required organized intracytoplasmic crystalline inclusions in proximal tubular epithelial cells. For non-crystalline LCPT, supportive proximal tubular ultrastructural abnormalities in the absence of crystals were required as a minimum, together with proximal tubular light-chain restriction or otherwise demonstrable monoclonal light-chain accumulation. When the ultrastructural abnormalities were non-specific, excess monoclonal urinary light chains and otherwise unexplained renal dysfunction were additionally required, consistent with the diagnostic framework of Kousios et al. (7). Cases dominated by cast nephropathy without supportive proximal tubular ultrastructural evidence were excluded. Conference abstracts without a full-text publication were excluded because they did not provide sufficient clinicopathological information for reproducible case-level verification and data extraction. Cases were also excluded if renal involvement or the underlying hematological diagnosis lacked pathological or bone marrow confirmation, or if the report was a duplicate publication.

#### 3.1.4 Data extraction

For each eligible record, we extracted age, sex, underlying hematological disease, monoclonal immunoglobulin/light-chain information, renal presentation, crystal distribution, treatment, and outcome, independently by two reviewers with discrepancies resolved by consensus; cohort-level reports were included only where WM/LPL status was confirmable at the individual-patient level.

#### 3.1.5 Registration

This review was not registered and no protocol was prepared.

### 3.2 Search results and case synthesis

Of 203 records identified (PubMed 27, Embase 40, and Web of Science 136; 155 after deduplication), 27 underwent full-text review following title/abstract screening, yielding 4 eligible studies, each contributing one case. Three additional eligible studies were identified through other methods: Kapadia et al. (15) and Bridoux et al. (16) through reference-list screening, and Kousios et al. (7) through manual review of an additional relevant full-text article and its supplementary patient-level data (**Supplementary Figure S1**). In total, seven eligible published cases were identified; including the present case, the final series comprises eight cases (**Table 2**).

**Table 2:** Published cases of renal light-chain proximal tubulopathy (LCPT) and/or crystal-storing histiocytosis (CSH) in Waldenström macroglobulinemia and IgM-secreting lymphoplasmacytic lymphoma identified by the systematic review (seven published cases; eight cases including the present case).

#	Source	Age/sex	Monoclonal Ig/LC	Hematological disease	Renal presentation	Renal lesion/location	Treatment	Outcome
1	Kapadia 1993 *Am J Surg Pathol* (Case 1 of 3) (15)	78/F	IgM-κ	LPL (not formally classified as WM in the original report)	AKI	Interstitial histiocytes (CSH); tubular involvement not assessable (no glomeruli/tubules in biopsy)	Chemotherapy	Died of AKI, 5 mo
2	Bridoux 2005 *Am J Kidney Dis* (16)	67/M	IgM-κ (Vκ3)	WM	Fanconi syndrome + CKD	Proximal tubules (LCPT, non-crystalline)	Chemotherapy (fludarabine)	Partial renal recovery
3	Ugai 2013 *Br J Haematol* (18)	76/M	IgM free-κ	WM	Fanconi syndrome	Proximal tubules (κ-LCPT)	Bortezomib + rituximab + dexamethasone (BRD)	Hematologic response (FLC/IgM↓) but metabolic tubular defects persisted; died of necrotizing fasciitis/multiple organ failure after the 2nd BRD cycle
4	Gupta 2019 *Ann Diagn Pathol* (13)	71/M	IgM-κ (κ/λ FLC ratio 25)	WM/LPL (17-year history)	Nephrotic-range proteinuria (3.6 g/day), mild renal impairment	Predominantly glomerular capillary loops (CD68-confirmed histiocytes) + proximal tubules + scattered interstitial histiocytes; lattice-structured crystals	Prior chemotherapy (multiple regimens) + splenectomy for relapse	Died of disease
5	Kang 2023 *Int Urol Nephrol* (17)	65/M	IgM-κ	WM (MYD88-L265P+)	Elevated creatinine, foamy urine, anemia; mild chronic tubulointerstitial damage	Proximal tubules—non-crystalline κ-LCPT (EM-confirmed absence of crystals, increased lysosomes)	NR	NR
6	Vos 2016 *Br J Haematol*, cohort case ‡ (3)	NR	NR (proximal tubular κ-light-chain staining)	WM	Sub-nephrotic proteinuria; no electrolyte/acid–base abnormality (i.e., no overt Fanconi syndrome)	Proximal tubules—”crystal-storing tubulopathy” with κ-light-chain staining; presumed crystalline LCPT‡‡	NR	NR
7	Kousios 2023 *Nephrol Dial Transplant* (Case 12) (7)	75/M	IgM-κ; κ-restricted LCPT	WM	CKD; eGFR 38 mL/min/1.73 m^2^; uPCR 111 mg/mmol	Proximal tubules—crystalline LCPT (EM inclusions present)	Dexamethasone–rituximab–cyclophosphamide (DRC)	Very good partial response; alive at 90 mo; eGFR +23%; uPCR −90%
8	Present case	65/M	IgM-κ	WM (MYD88-L265P+)	Progressive CKD; IgA nephropathy pattern; discordant MPO-ANCA seropositivity	Proximal tubules (crystalline κ-LCPT confirmed by EM) + interstitial mononuclear cells (ultrastructural; CSH unconfirmed—no CD68/CD163)	Sequential BTKi (ibrutinib → zanubrutinib) + temporary bortezomib–dexamethasone add-on	Stable at 49 mo; IgM 13.8→3.9 g/L; eGFR 41.8→44.2 mL/min/1.73 m^2^; later 24-h protein unavailable

‡Cohort-derived subgroup case(s): individual-level age, sex, and case-specific treatment/outcome were not separately reported in the source publication, which presents only pooled or subgroup-level data (see Data extraction, Methods). ‡‡Original report uses the term “crystal-storing tubulopathy” rather than LCPT or CSH; based on the description (κ-light-chain staining within proximal tubular epithelial cells, no mention of histiocytic involvement), this most likely represents crystalline LCPT under current nomenclature. LCPT, light-chain proximal tubulopathy; CSH, crystal-storing histiocytosis; BTKi, BTK inhibitor; DRC, dexamethasone–rituximab–cyclophosphamide; EM, electron microscopy; FS, Fanconi syndrome; LPL, lymphoplasmacytic lymphoma; MGRS, monoclonal gammopathy of renal significance; NR, not reported; WM, Waldenström macroglobulinemia. For the Vos et al. cohort-derived case, the light-chain isotype of the serum monoclonal IgM was not reported at the individual-patient level; κ refers to κ-light-chain staining within proximal tubular epithelial cells.

Bridoux et al. (16) described WM-associated LCPT in a man with full-blown Fanconi syndrome and monoclonal Vκ3 light-chain accumulation in proximal tubules without crystalline organization, treated with chemotherapy with partial renal recovery. Kang et al. (17) described EM-confirmed non-crystalline LCPT with IgM free κ in MYD88-L265P-positive WM, with no treatment or outcome reported. Ugai et al. (18) reported renal Fanconi syndrome from monoclonal κ free light chain in WM, with EM-confirmed crystalline LCPT on biopsy. Kousios et al. (7) reported a 75-year-old man with WM and IgM-κ crystalline LCPT (case 12 of their cohort); after DRC therapy, he achieved a very good partial response and remained alive at 90 months, with a 23% increase in eGFR and 90% reduction in uPCR. Kapadia et al. (15) reported three cases of CSH associated with lymphoplasmacytic neoplasms; the one IgM-κ case involved a 78-year-old woman whose renal biopsy showed parenchyma replaced by crystal-storing histiocytes, precluding assessment of LCPT, and who died of acute renal failure approximately 5 months after presentation. Gupta et al. (13) (case 1 of three) documented a 71-year-old man with WM/LPL and IgM-κ CSH predominantly involving glomerular capillary loops, with CD68-confirmed proximal tubular and interstitial histiocytic involvement. Vos et al. (3), in a cohort of 44 WM-related nephropathy biopsies, identified one case of “crystal-storing tubulopathy” with κ-light-chain staining in proximal tubules, most consistent with crystalline LCPT under current nomenclature, though individual-level age, sex, treatment, and outcome were not reported.

Taken together, LCPT was identified in five of the seven published cases [three crystalline/probable crystalline: Ugai et al. (18), Kousios et al. (7), and Vos et al. (3); two non-crystalline: Bridoux et al. (16) and Kang (17)], and CSH was identified in two cases [Kapadia et al. (15) and Gupta et al. (13)]. IgM-κ was reported in the individually characterized published cases with available paraprotein data; for the Vos et al. cohort-derived case, the individual serum IgM light-chain isotype was not reported, although κ-light-chain staining was present in proximal tubules. Among the six cases with individually reported age and sex, ages ranged from 65 to 78 years with a male predominance (five of six). Outcomes were heterogeneous: Bridoux et al. (16) reported partial renal recovery, while case 12 of Kousios et al. achieved a very good partial response with improved eGFR and marked reduction in proteinuria (7); other reports described persistent tubular abnormalities, death, or unavailable outcomes. None of the published cases received Bruton tyrosine kinase (BTK) inhibitor therapy.

## 4 Discussion

### 4.1 The present case in the context of the literature review

This systematic review and case together indicate that crystalline κ-LCPT and CSH are among the rarest renal manifestations of WM. Against this background, the present case is distinctive in three respects (1): it adds to the small published series of EM-confirmed crystalline κ-LCPT in WM, meeting the diagnostic criteria for this entity (2); it demonstrates concurrent ultrastructural extra-tubular crystalline deposition raising—but, in the absence of CD68/CD163 immunostaining, not confirming—the possibility of CSH; and (3) it occurs in MYD88-L265P-mutated WM with two additional diagnostic complexities: a concurrent IgA nephropathy pattern and MPO-ANCA seropositivity that was discordant with the renal biopsy. No prior case in the reviewed literature combined all three features.

### 4.2 κ-LCPT in WM: a rare entity with a distinctive presentation in this case

LCPT occurs predominantly in plasma-cell dyscrasias and MGRS and is distinctly uncommon in WM (9, 19–21). Whereas many glomerular WM lesions reflect circulating IgM-containing immune complexes or monoclonal deposits, tubular crystal formation depends on intrinsic structural features of the κ-light-chain variable domain—including restriction to specific germline subgroups and hydrophobic substitutions within the complementarity-determining regions—which promote self-aggregation into ordered crystalline inclusions (20). Light chains lacking these features are not necessarily inert, however: one recognized non-crystalline pathway involves incomplete catabolism of the light chain within markedly enlarged, “indigested” lysosomes, which can produce a Fanconi-type phenotype indistinguishable from crystalline disease despite the absence of any crystalline inclusion (22).

Notably, crystal formation itself does not appear to reliably determine clinical phenotype. Among the WM cases reviewed here, Fanconi syndrome occurred in only two of the six LCPT cases considered here: the crystalline case reported by Ugai et al. (18) and the non-crystalline case reported by Bridoux et al. (16). The remaining cases, including the present one, showed no clinical evidence of Fanconi syndrome despite κ-light-chain accumulation in proximal tubular epithelial cells. A similar dissociation has been observed even within crystalline disease alone: in a recent single-center series, Fanconi syndrome was present in only 10% of patients with LCPT overall, occurring exclusively among crystalline cases, yet in a smaller proportion than previously reported, leading the authors to propose that the mechanisms driving Fanconi syndrome may be independent of the crystal formation process itself (21).

Consistent with the present case, crystalline inclusions can be inconspicuous or entirely inapparent on light microscopy: the cytoplasmic inclusions typical of crystalline LCPT are often PAS-negative and easily overlooked as subtle, ill-defined cleftlike spaces (22), and in the largest dedicated LCPT cohort, crystals were undetectable by light microscopy and identified only by EM in 5 of 38 crystalline cases (9). Kousios et al. further showed that crystalline LCPT may display rhomboid or spicular organized structures that are either electron-dense or electron-lucent, whereas non-crystalline LCPT shows a broad spectrum of fibrillary, fibrillogranular, granular, vacuolar, or abnormal lysosomal ultrastructural changes (7). In the same series, semi-thin toluidine blue-stained sections prepared for EM demonstrated rhomboid and/or spicular crystals in four of five crystalline cases, and protease-digested paraffin immunofluorescence demonstrated light-chain restriction or predominance when frozen-section staining showed no obvious restriction; EM remained the key method for defining the ultrastructural lesion (7). In the present case, both frozen-section and pepsin-digested paraffin-section light-chain immunofluorescence showed tubular κ predominance while glomerular κ/λ staining remained non-restricted (**Figure 2B**).

### 4.3 Ultrastructural crystal deposition in interstitial mononuclear cells

Ultrastructural rhomboid crystalline deposits were identified within interstitial mononuclear cells by EM, raising the possibility of CSH. To evaluate for lymphomatous renal infiltration, repeat pepsin-digested paraffin-section light-chain immunofluorescence and an extended immunohistochemical panel were performed. The interstitial infiltrate contained CD5-positive and CD20-positive cells without evidence of a monotypic B-cell population, arguing against direct lymphomatous infiltration of the renal parenchyma. Focal interstitial mononuclear cells showed cytoplasmic κ predominance, and transmission EM demonstrated rhomboid crystalline material within the same anatomical compartment (**Figure 3D**). Residual tissue was unavailable for additional CD68/CD163 immunostaining; therefore, histiocytic lineage could not be confirmed, and the finding was considered suggestive of, but not diagnostic of, CSH. CSH is well recognized to be associated with a wide range of lymphoplasmacytic and plasma cell neoplasms (23, 24), but its distribution across these is highly uneven: in the largest pooled literature review to date (181 cases across all anatomic sites globally), multiple myeloma accounted for 34.1% of associated neoplastic conditions, marginal zone lymphoma accounted for 25.4%, LPL accounted for 14.5%, and MGUS accounted for 11.6%, whereas WM accounted for only 6 cases (4.35% of neoplastic associations) (24). Notably, of the six WM-associated CSH cases captured in that review, only one involved the kidney—corresponding to Gupta et al. (13), already included in our systematic review (**Table 2**, case 4). This rarity is consistent with the paucity of WM-specific cases identified in our systematic review.

### 4.4 IgA nephropathy pattern in the setting of WM

The glomerular lesion showed an IgA nephropathy pattern characterized by strong mesangial IgA (+++) and C3 (++) deposition, polytypic glomerular κ- and λ-light-chain staining without restriction, and corresponding predominantly mesangial electron-dense deposits (**Figures 1B, C**). Light-microscopic mesangial proliferative changes were relatively mild (**Figure 1A**), suggesting that the IgA nephropathy pattern lesion represented a concurrent but comparatively limited glomerular component in the current biopsy. This polytypic glomerular pattern contrasted with the κ-predominant crystalline proximal tubular lesion. Importantly, the circulating monoclonal immunoglobulin was IgM-κ, whereas the glomerular deposits were IgA-dominant and lacked κ restriction, arguing against direct deposition of the WM-derived monoclonal immunoglobulin as the mechanism of the glomerular lesion. This distinction is relevant because directly monoclonal IgA-associated glomerular lesions generally demonstrate monotypic immunoglobulin/light-chain features (25, 26), whereas conventional IgA nephropathy is an immune-complex disease with heterogeneous IgA-containing complexes (27). Coexistence of monoclonal gammopathy with kidney diseases unrelated to the monoclonal protein is well documented: in a large kidney-biopsy cohort of patients with monoclonal gammopathy, 62% had non-MGRS lesions and IgA nephropathy accounted for 14% of that group (28). Individual reports have likewise described IgA nephropathy occurring alongside IgM monoclonal gammopathy (29) and IgG-κ multiple myeloma (30). Nevertheless, the present biopsy cannot establish whether the IgA-pattern glomerular lesion was entirely independent of WM. The preceding intermittent hematuria and proteinuria may indicate a longer-standing IgA-mediated glomerular process, but an indirect contribution from B-cell/immune dysregulation associated with WM cannot be excluded.

### 4.5 MPO-ANCA seropositivity without ANCA-associated glomerulonephritis

MPO-ANCA seropositivity initially raised concern for ANCA-associated vasculitis, but the serological and pathological findings were discordant. MPO-ANCA was strongly positive by qualitative EUROLINE immunoblotting (EUROIMMUN, Lübeck, Germany) on 3 March 2022 and remained modestly elevated at 37.86 RU/mL by HOB Biotech chemiluminescence on a separate sample on 21 March 2022, whereas P-ANCA and C-ANCA were negative by indirect immunofluorescence and PR3-ANCA was negative. True ANCA-positive IgA nephropathy/AAV overlap has been reported and typically presents with active necrotizing and/or crescentic glomerulonephritis, often with a more aggressive clinical phenotype (31–33). In contrast, our kidney biopsy showed no active pauci-immune necrotizing or cellular crescentic glomerulonephritis; the single crescent was fibrous, and the immune-complex IgA deposits provided an alternative explanation for the glomerular lesion. Monoclonal immunoglobulins can interfere with serological assays through non-specific binding, and paraproteinemia is recognized as a potential source of misleading autoantibody results (34–36). Accordingly, the MPO-ANCA result in this patient was considered likely to represent non-specific/paraprotein-associated assay reactivity rather than ANCA-mediated renal disease. MPO-ANCA was not retested during later follow-up, so direct demonstration of seroreversion or a specific interference mechanism was not available. The practical implication is that ANCA seropositivity in a patient with monoclonal gammopathy should be interpreted in conjunction with clinical findings and, when renal involvement is present, kidney biopsy before immunosuppression is initiated.

### 4.6 BTK inhibitors in WM-associated crystalline LCPT

Sequential BTK inhibitor therapy was associated with a major hematological response and stable renal function over 49 months of follow-up. This treatment course is biologically consistent with the established MYD88–interleukin-1 receptor-associated kinase (IRAK)1/4–BTK–nuclear factor κB (NF-κB) signaling framework of WM. MYD88-L265P promotes survival of the lymphoplasmacytic clone through IRAK1/4, BTK, and downstream NF-κB signaling. Experimental studies have demonstrated functional coupling of MYD88-L265P to activated BTK and shown that inhibition of BTK or IRAK1/4 promotes apoptosis of WM cells, providing a molecular rationale for BTK-directed therapy (37). BTK inhibitors are established therapies for MYD88-L265P-mutated WM; patients harboring this mutation, as in the present case, achieve a major response rate to ibrutinib of approximately 91%, substantially higher than that observed in MYD88-wild-type disease (14). More selective second-generation BTK inhibitors such as zanubrutinib offer improved tolerability compared with ibrutinib (14), and their potential role in WM-associated renal disease has also been highlighted recently (38).

In WM-associated LCPT, BTK inhibition targets the upstream pathogenic lymphoplasmacytic clone, thereby reducing ongoing monoclonal κ-light-chain production and proximal tubular exposure rather than directly removing pre-existing intracellular crystals. In the present patient, the marked reductions in serum IgM and the free κ/λ ratio, together with sustained renal stability, are consistent with reduced ongoing monoclonal light-chain burden. These observations connect clonal B-cell signaling, monoclonal κ-light-chain production, proximal tubular injury, and BTK-directed therapy within a coherent biological framework, while supporting the feasibility of BTK inhibitor-based clone-directed treatment for WM-associated crystalline LCPT.

### 4.7 Study limitations

This study has several limitations. First, residual renal tissue was unavailable for additional CD68/CD163 immunostaining, precluding definitive confirmation of histiocytic lineage and classification of the interstitial crystalline deposits as CSH. Second, although the biopsy established an IgA nephropathy pattern, its etiological relationship to WM remains uncertain; the polytypic glomerular deposits argue against direct deposition of the monoclonal IgM-κ protein. Third, the systematic review is constrained by the small number of published cases and predominantly case-report-level evidence.

## 5 Conclusions

This case demonstrates biopsy-confirmed crystalline κ-LCPT in MYD88-L265P-positive WM with a concurrent polytypic IgA nephropathy pattern and interstitial crystalline deposition suggestive of, but not diagnostic of, CSH. Kidney biopsy excluded active ANCA-associated glomerulonephritis despite MPO-ANCA seropositivity. The sustained hematological response with stable renal function over 49 months supports the feasibility of BTK inhibitor-based clone-directed therapy while underscoring the importance of integrating renal pathology with clonal B-cell evaluation.

## 6 Patient perspective

The patient was counseled about the diagnosis and the rationale for sequential BTK inhibitor therapy. He reported improved energy and wellbeing as his monoclonal protein levels fell, and tolerated treatment well over 4 years of follow-up. He expressed satisfaction with the stability of his kidney function and continues regular hematology and nephrology follow-up.

## Data Availability

The raw data supporting the conclusions of this article will be made available by the authors, without undue reservation.

## References

[1] Uppal NN Monga D Vernace MA Mehtabdin K Shah HH Bijol V . Kidney diseases associated with Waldenström macroglobulinemia. Nephrol Dial Transplant. (2019) 34:1644–52. doi: 10.1093/ndt/gfy320 30380110

[2] Gertz MA . Waldenström macroglobulinemia: 2025 update on diagnosis, risk stratification, and management. Am J Hematol. (2025) 100:1061–73. doi: 10.1002/ajh.27666 40095219

[3] Vos JM Gustine J Rennke HG Hunter Z Manning RJ Dubeau TE . Renal disease related to Waldenström macroglobulinaemia: incidence, pathology and clinical outcomes. Br J Haematol. (2016) 175:623–30. doi: 10.1111/bjh.14279 27468978

[4] Chauvet S Bridoux F Ecotière L Javaugue V Sirac C Arnulf B . Kidney diseases associated with monoclonal immunoglobulin M-secreting B-cell lymphoproliferative disorders: a case series of 35 patients. Am J Kidney Dis. (2015) 66:756–67. doi: 10.1053/j.ajkd.2015.03.035 25987261

[5] Higgins L Nasr SH Said SM Kapoor P Dingli D King RL . Kidney involvement of patients with Waldenström macroglobulinemia and other IgM-producing B cell lymphoproliferative disorders. Clin J Am Soc Nephrol. (2018) 13:1037–46. doi: 10.2215/CJN.13041117 29848505 PMC6032586

[6] Audard V Georges B Vanhille P Toly C Deroure B Fakhouri F . Renal lesions associated with IgM-secreting monoclonal proliferations: revisiting the disease spectrum. Clin J Am Soc Nephrol. (2008) 3:1339–49. doi: 10.2215/CJN.01600408 18632851 PMC2518806

[7] Kousios A Blakey S Moran L Atta M Charif R Duncan N . Non-crystalline light chain proximal tubulopathy, a morphologically protean entity. Nephrol Dial Transplant. (2023) 38:2576–88. doi: 10.1093/ndt/gfad085 37120733 PMC10615624

[8] Sanders PW . Mechanisms of light chain injury along the tubular nephron. J Am Soc Nephrol. (2012) 23:1777–81. doi: 10.1681/ASN.2012040388 22997259

[9] Stokes MB Valeri AM Herlitz L Khan AM Siegel DS Markowitz GS . Light chain proximal tubulopathy: clinical and pathologic characteristics in the modern treatment era. J Am Soc Nephrol. (2016) 27:1555–65. doi: 10.1681/ASN.2015020185 26374607 PMC4849818

[10] Ma CX Lacy MQ Rompala JF Dispenzieri A Rajkumar SV Greipp PR . Acquired Fanconi syndrome is an indolent disorder in the absence of overt multiple myeloma. Blood. (2004) 104:40–2. doi: 10.1182/blood-2003-10-3400 15010372

[11] Luciani A Sirac C Terryn S Javaugue V Prange JA Bender S . Impaired lysosomal function underlies monoclonal light chain-associated renal Fanconi syndrome. J Am Soc Nephrol. (2016) 27:2049–61. doi: 10.1681/ASN.2015050581 26614382 PMC4926980

[12] El Hamel C Thierry A Trouillas P Bridoux F Carrion C Quellard N . Crystal-storing histiocytosis with renal Fanconi syndrome: pathological and molecular characteristics compared with classical myeloma-associated Fanconi syndrome. Nephrol Dial Transplant. (2010) 25:2982–90. doi: 10.1093/ndt/gfq129 20356978

[13] Gupta RK Rosenberg AZ Bagnasco SM Arend LJ . Renal crystal-storing histiocytosis involving glomeruli - a comprehensive clinicopathologic analysis. Ann Diagn Pathol. (2019) 43:151403. doi: 10.1016/j.anndiagpath.2019.151403 31494491

[14] Patel N Al Hadidi S Yellapragada S . Pathophysiology and treatments of complications of Waldenström’s macroglobulinemia. Clin Hematol Int. (2024) 6:11–8. doi: 10.46989/001c.124268 39417016 PMC11477924

[15] Kapadia SB Enzinger FM Heffner DK Hyams VJ Frizzera G . Crystal-storing histiocytosis associated with lymphoplasmacytic neoplasms. Report of three cases mimicking adult rhabdomyoma. Am J Surg Pathol. (1993) 17:461–7. doi: 10.1097/00000478-199305000-00004 8470760

[16] Bridoux F Sirac C Hugue V Decourt C Thierry A Quellard N . Fanconi’s syndrome induced by a monoclonal Vkappa3 light chain in Waldenstrom’s macroglobulinemia. Am J Kidney Dis. (2005) 45:749–57. doi: 10.1053/j.ajkd.2004.12.020 15806478

[17] Kang T Tang D Mao H Liu Q Gan L Liu J . Non-crystalline light chain proximal tubular disease associated with Waldenström macroglobulinemia. Int Urol Nephrol. (2023) 55:483–5. doi: 10.1007/s11255-022-03304-3 35859221

[18] Ugai T Tsuda K Sugihara H Nishida Y Yamakura M Takeuchi M . Renal Fanconi syndrome associated with monoclonal κ free light chain in a patient with Waldenström macroglobulinemia. Br J Haematol. (2013) 162:1. doi: 10.1111/bjh.12398 23758066

[19] Larsen CP Bell JM Harris AA Messias NC Wang YH Walker PD . The morphologic spectrum and clinical significance of light chain proximal tubulopathy with and without crystal formation. Mod Pathol. (2011) 24:1462–9. doi: 10.1038/modpathol.2011.104 21701535

[20] Sirac C Batuman V Sanders PW . The proximal tubule toxicity of immunoglobulin light chains. Kidney Int Rep. (2021) 6:1225–31. doi: 10.1016/j.ekir.2021.02.026 34013100 PMC8116766

[21] Wang X Yu XJ Wang SX Zhou FD Zhao MH . Light-chain proximal tubulopathy: a retrospective study from a single Chinese nephrology referral center. Ren Fail. (2024) 46:2283587. doi: 10.1080/0886022X.2023.2283587 PMC10880565 38374684

[22] Herrera GA . Proximal tubulopathies associated with monoclonal light chains: the spectrum of clinicopathologic manifestations and molecular pathogenesis. Arch Pathol Lab Med. (2014) 138:1365–80. doi: 10.5858/arpa.2013-0493-OA 25268200

[23] Reeders J Arnold C Chen J Kirwan P Lynnhtun K . Crystal-storing histiocytosis leading to the identification of IgG-kappa secreting lymphoplasmacytic lymphoma with crystalline nephropathy. Pathology. (2020) 52:283–6. doi: 10.1016/j.pathol.2019.11.004 31883675

[24] Mobarki M Papoudou-Bai A Dumollard JM Alhazmi AH Musawi S Madkhali MA . Crystal-storing histiocytosis: the iceberg of more serious conditions. Diagn (Basel). (2023) 13:271. doi: 10.3390/diagnostics13020271 36673081 PMC9858286

[25] Li J-P Du Y-T Guo C Rao X-R Li S . IgA nephropathy to proliferative glomerulonephritis with monoclonal IgAκ deposits: a pattern switch. J Nephrol. (2023) 36:2375–80. doi: 10.1007/s40620-023-01583-2 36913081 PMC10638190

[26] Li X-Y Xie G-L Chen B Zhang J Chen J-S Ding X-K . IgA nephropathy with monotypic IgA-κ deposits: a case report and literature review. Front Med (Lausanne). (2025) 12:1643630. doi: 10.3389/fmed.2025.1643630 41195171 PMC12583212

[27] Knoppova B Reily C Maillard N Rizk DV Moldoveanu Z Mestecky J . The origin and activities of IgA1-containing immune complexes in IgA nephropathy. Front Immunol. (2016) 7:117. doi: 10.3389/fimmu.2016.00117 27148252 PMC4828451

[28] Yong Z-H Yu X-J Liu J-X Zhou F Wang S-X Zhao M-H . Kidney histopathologic spectrum and clinical indicators associated with MGRS. Clin J Am Soc Nephrol. (2022) 17:527–34. doi: 10.2215/CJN.12890921 35210280 PMC8993475

[29] Wu H-T Wen Y-B Ye W Liu B-Y Shen K-N Gao R-T . Underlying IgM heavy chain amyloidosis in treatment-refractory IgA nephropathy: a case report. World J Clin cases. (2019) 7:3055–61. doi: 10.12998/wjcc.v7.i19.3055 31624754 PMC6795715

[30] Couto ME Sousa D Ferreira H Tavares A Oliveira I Domingues N . IgA nephropathy in IgG kappa multiple myeloma. Porto BioMed J. (2021) 6:e142. doi: 10.1097/j.pbj.0000000000000142 34651090 PMC8509915

[31] Torres Ruiz AC Ruiz Ravelo RM Ruiz Cruz NC Fernández Rivera JDJ De Lira PKH . IgA nephropathy associated with ANCA vasculitis: case report and literature review. IJMSCRS. (2023) 03:2894–9. doi: 10.47191/ijmscrs/v3-i11-66

[32] Chogle AS Suresh S Gunabooshanam B Elumalai R Haridas A . IgA nephropathy with ANCA vasculitis: a case section with diagnostic and therapeutic challenges. JCDR. (2026) 20(5):OD06–8. doi: 10.7860/JCDR/2026/81059.23217

[33] Haas M Jafri J Bartosh SM Karp SL Adler SG Meehan SM . ANCA-associated crescentic glomerulonephritis with mesangial IgA deposits. Am J Kidney Dis. (2000) 36:709–18. doi: 10.1053/ajkd.2000.17615 11007672

[34] Kuroda K Nakazaki H Kosaka H Hoshio H . False-positive myeloperoxidase-antineutrophil cytoplasmic antibody in a patient with rheumatoid arthritis. Am J Case Rep. (2023) 24:e941306. doi: 10.12659/AJCR.941306 37752696 PMC10546948

[35] Gandhi S Little MA Farrington K . Waldenstrom’s macroglobulinemia presenting as Goodpasture’s syndrome. Ren Fail. (2004) 26:591–6. doi: 10.1081/jdi-200031715 15526921

[36] Moiseev S Cohen Tervaert JW Arimura Y Bogdanos DP Csernok E Damoiseaux J . 2020 international consensus on ANCA testing beyond systemic vasculitis. Autoimmun Rev. (2020) 19:102618. doi: 10.1016/j.autrev.2020.102618 32663621

[37] Yang G Zhou Y Liu X Xu L Cao Y Manning RJ . A mutation in MYD88 (L265P) supports the survival of lymphoplasmacytic cells by activation of Bruton tyrosine kinase in Waldenström macroglobulinemia. Blood. (2013) 122:1222–32. doi: 10.1182/blood-2012-12-475111 23836557

[38] Giachetti R Ravaglia F Somma C Mannucci C Scaramuzzino F Rousseau M . Not just amyloidosis: the spectrum of Waldenstrom’s macroglobulinemia-associated renal disease. Leuk Lymphoma. (2025) 66:1980–9. doi: 10.1080/10428194.2025.2512392 40488737

