# Supplementary material for: Crystalline κ-light-chain proximal tubulopathy in Waldenström macroglobulinemia with an IgA nephropathy pattern: a case report and systematic review
Source: Front Immunol. 2026 Oct 15;17:1942324. doi: 10.3389/fimmu.2026.1942324 (PMC13716615; doi:10.3389/fimmu.2026.1942324)
Supplement: Supplementary file 1 [file DataSheet1.docx]

**Supplementary Material**

Crystalline κ-Light Chain Proximal Tubulopathy in Waldenström Macroglobulinemia with an IgA Nephropathy Pattern: A Case Report and Systematic Review

**Supplementary Figure S1. PRISMA 2020 study selection flow diagram.**

Records identified through database searching were PubMed/MEDLINE 27, Embase 40, and Web of Science 136 (total n=203; n=155 after removal of 48 duplicates). Title/abstract screening excluded 128 records (non-WM/LPL background n=80, review articles without original cases n=28, conference abstracts n=8, animal studies n=2, no renal biopsy n=4, and no renal involvement n=6), leaving 27 reports for full-text review. Full-text review excluded 23 reports (renal biopsy did not report LCPT or CSH pathology n=12; non-WM/IgM-LPL at full-text reassessment n=10; and cohort-level LCPT/CSH status not confirmable at the individual-patient level n=1), leaving 4 eligible studies from the database search (Ugai 2013; Gupta 2019; Kang 2023; Vos 2016). Three additional eligible studies were identified through other methods: Kapadia 1993 and Bridoux 2005 through reference-list screening, and Kousios 2023 through manual review of an additional relevant full-text article and its supplementary patient-level data. Each of the 7 eligible published studies contributed one case meeting the review criteria. In Vos 2016, the relevant LCPT case was reported within pooled cohort data rather than as a separate case description and was incorporated into Table 2 accordingly. Including the present case, 8 cases were included in the final synthesis.
